# COVID-19 was associated with the complications after allogeneic hematopoietic stem cell transplantation

**DOI:** 10.1038/s41598-024-62731-7

**Published:** 2024-05-23

**Authors:** Qi Wen, Ze Guo, Xiao-Hui Zhang, Lan-Ping Xu, Yu Wang, Chen-Hua Yan, Huan Chen, Yu-Hong Chen, Wei Han, Feng-Rong Wang, Yu-Qian Sun, Xiao-Jun Huang, Xiao-Dong Mo

**Affiliations:** 1 Beijing Key Laboratory of Hematopoietic Stem Cell Transplantation, National Clinical Research Center for Hematologic Disease, Peking University People’s Hospital, Peking University Institute of Hematology, No. 11 Xizhimen South Street, Xicheng District, Beijing, 100044 China; 2 Research Unit of Key Technique for Diagnosis and Treatments of Hematologic Malignancies, Chinese Academy of Medical Sciences, Beijing, 2019RU029 China

**Keywords:** Diseases, Medical research

## Abstract

We aimed to identify the severity and duration of COVID-19 infection on complications after allo-HSCT. Enrolled 179 hospitalized patients with COVID-19 were categorized into long-term infection (> 18 days, *n* = 90) or short-term infection group (≤ 18 days, *n* = 89) according to the median duration of COVID-19. The severity of COVID-19 was categorized as asymptomatic infection, mild, moderate, severe, and critical illness according to guidelines of National Institutes of Health. Particularly, severe illness and critical illness were classified as serious infection. Asymptomatic infection, mild illness and moderate illness were classified as non-serious infection. The 150-day probabilities of poor graft function (PGF), cytomegalovirus (CMV) pneumonia and non-relapse mortality (NRM) were significantly higher in long-term infection group. The 150-day probabilities of CMV pneumonia and NRM after COVID-19 were higher in serious infection group. The 150-day probabilities of overall survival (OS) was significantly lower in long-term and serious infection group. In multivariable analysis, the severity of COVID-19 was associated with NRM and OS, and the duration of COVID-19 was associated with PGF. In summary, our data reported that the severity and duration of COVID-19 were associated with several complications and contribute to poor outcomes after allo-HSCT.

## Introduction

Allogeneic hematopoietic stem cell transplantation (allo-HSCT) provides a potential curative therapy for patients with both malignant and nonmalignant hematological disease^1,2^. Nevertheless, infections, particularly the viral infection, remain one of the most life-threatening complications after allo-HSCT^3,4^.

Severe acute respiratory syndrome coronavirus 2 (SARS-CoV-2) caused Coronavirus Disease 2019 (COVID-19) since the end of 2019 and had caused over 2 million deaths worldwide^5,6^. COVID-19 has also become one of the important viral infections in allo-HSCT recipients^7,8^. Since November 2021, the appearance of Omicron variant and its sublineages further worse the epidemiological situation. Although the lower replication competence of Omicron in the human lungs^9,10^ may cause lower the disease severity^11–13^, the Omicron variant pandemic in Hong Kong from January to March 2022 still cause a COVID-19 mortality rate of 37.7 per million population^14^. Thus, we should not underestimate the harm of Omicron variant infection in allo-HSCT recipients.

Virus could cause several serious complications after allo-HSCT. For example, virus is the most common pathogen of the late-onset severe pneumonia after allo-HSCT and the mortality rate could be as high as 60%^15^. Besides of the direct pathogenesis and injury, viral infections are also associated with several common complications after allo-HSCT. For example, parvovirus B19 infection could cause secondary poor graft function (PGF) and graft rejection^16^. In addition, herpes virus, particularly the human herpesvirus 6 and cytomegalovirus (CMV), is associated with the occurrence of acute graft-versus-host disease (aGVHD)^17–19^. Thus, we speculate that SARS-CoV-2 may also contribute to the occurrence of post-transplant complications.

Although several studies reported COVID-19 infection in allo-HSCT recipients, most of them only focused on the signs, symptoms, prognostics factors, and the treatments and they only reported the characteristics of COVID-19 before 2021 which might be not the same as the Omicron variants. In the Omicron era, several studies have identified the characteristics of COVID-19 in immunocompromised patients, but most of them also only focused on the clinical presentations and treatments of COVID-19, and the sample of allo-HSCT recipients was small^7,8,20–22^. Thus far, no study had focused on the association between occurrence, severity, and duration of COVID-19 infection and complications after allo-HSCT. Particularly, the influence of Omicron variant infection on the post-transplant complications is unclear.

Thus, we aimed to identify the severity and duration of COVID-19 on post-transplant complications in the Omicron era.

## Results

### Patients’ characteristics

The characteristics of consecutive 179 hospitalized COVID-19 patients were showed in Table 1. The comparison between serious and non-serious infection group was showed in Supplementary Table 1. Forty-three (24%) and 136 (76%) patients were diagnosed as serious and non-serious infection, respectively. The median duration of disease was 18 days (range 2–102) days, and duration of SARS-CoV-2 infection > 18 days and ≤ 18 days was defined as long-term (*n* = 90) and short-term infection (*n* = 89), respectively (Supplementary Table 2). The median time from transplantation to COVID-19 infection occurrence was 149 days (range 6–3038) days. A total of 116 (64.8%) patients received immunosuppressants, including cyclosporin (*n* = 85), tacrolimus (*n* = 8), ruxolitinib (*n* = 8), or glucocorticoids (*n* = 32) when COVID-19 occurrence. Seventeen patients received more than 1 type of immunosuppressants. A total of 144 (80.4%) patients received anti-viral treatment (140 for Paxlovid and 4 for Azvudine). Forty serious infection cases (93.0%) received anti-viral treatment (39 for Paxlovid and 1 for Azifudine), 85 long-term infection cases (94.4%) received anti-viral treatment (83 for Paxlovid and 2 for Azifudine). Sixty-two patients received newly added drugs corticosteroid treatment, that is, 23 (53.5%) and 39 (28.7%) patients were in the serious and non-serious infection group, 9 (10.1%) and 53 (58.9%) patients were in the short- and long-term infection group, respectively.

**Table 1:** Characteristics of allo-HSCT recepient.

Characteristics	COVID-19 (n = 179)
Gender, male/female, n (%)	108 (60.3)/71 (39.7)
Age(years), range	40 (5–75)
Underlying disease, n (%)	
AL	131 (73.2)
MDS	24 (13.4)
AA	5 (2.8)
Lymphoma	10 (5.6)
Others	9 (5.0)
Donor match, n (%)	
HLA-matched sibling donor	31 (17.3)
HLA-matched unrelated donor	6 (3.4)
Haploidentical related donor	142 (79.3)
Blood group matched, n (%)	
Matched	103 (57.5)
Minor mismatched	30 (16.8)
Major mismatched	46 (25.7)
HCT-CI before HSCT, n (%)	
0 (low risk)	123 (68.7)
1–2 (intermediate risk)	41 (22.9)
≥ 3 (high risk)	15 (8.4)
Median counts of MNC in graft, range (× 10^8^/kg)	9.51 (3.83–19.90)
Median counts of CD34^+^ cell in graft, range (× 10^6^/kg)	3.08 (0.48–17.20)
Conditioning, n (%)	
Chemotherapy-based	168 (93.9)
TBI-based	11 (6.1)
Median counts of lymphocytes at COVID-19 diagnosis, range (× 10^9^/L)	1.00 (0.00–6.21)
COVID-19, n (%)	
Asymptomatic infection	14 (7.8)
Mild illness	78 (43.6)
Moderate illness	44 (24.6)
Severe illness	22 (12.3)
Critical illness	21 (11.7)

*AL* acute leukemia, *MDS* myelodysplastic syndromes, *AA* aplastic anemia, *HLA* human leukocyte antigen, *HCT-CI* hematopoietic cell transplantation–specific comorbidity index, *MNC* mononuclear cell, *TBI* total body irradiation.

### The severity and duration of COVID-19 and PGF

A total of 26, 33, and 11 patients showed PGF, leukopenia and thrombocytopenia, respectively, after COVID 19 infection. The 150-day cumulative incidence of leukopenia, thrombocytopenia, and PGF was 18.4% (95% confidence interval [CI] 12.7–24.1%), 6.1% (95% CI 2.6–19.6%), and 14.5% (95% CI 9.3–19.7%) respectively, after COVID-19.

**Figure 1:**
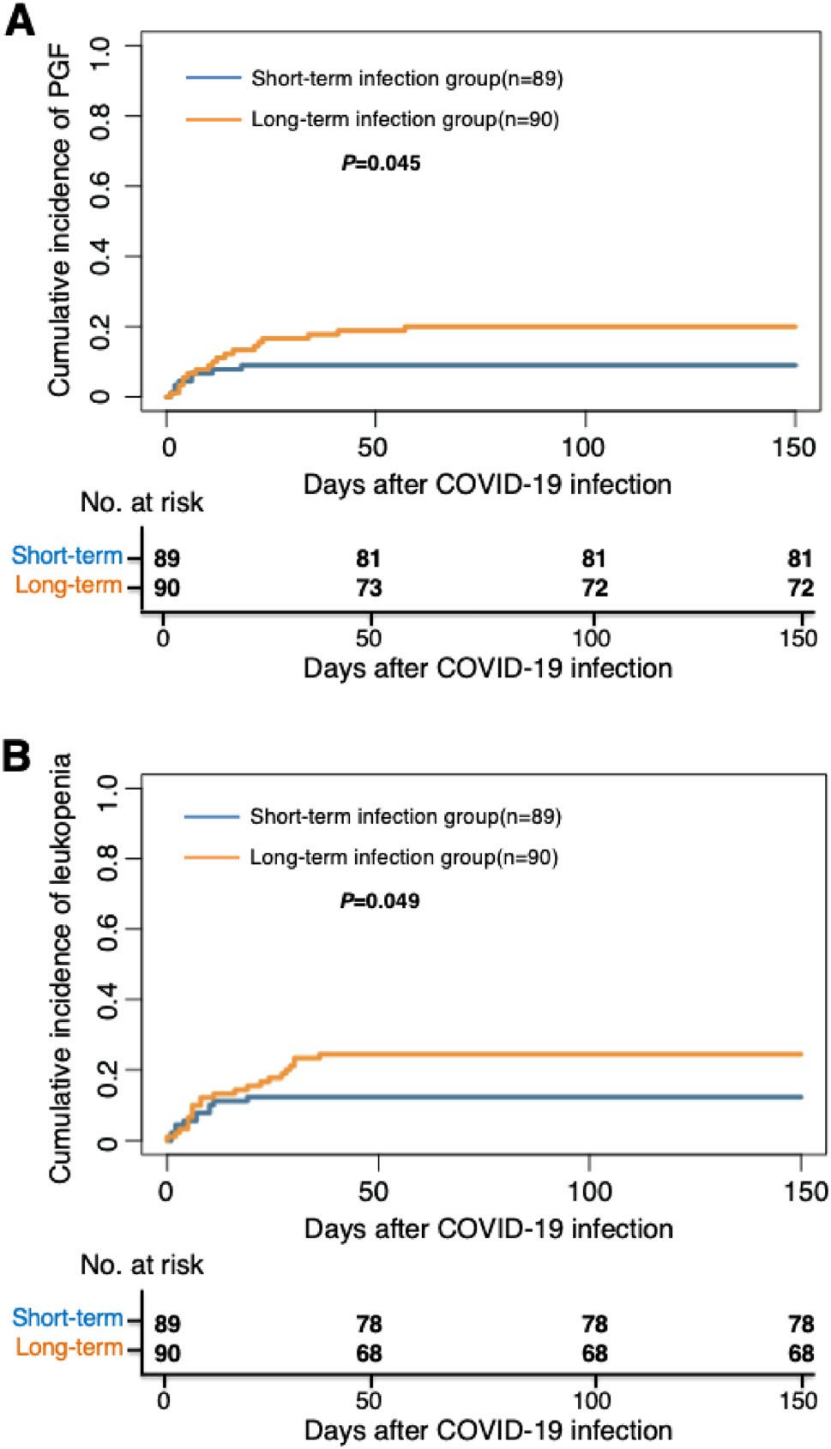
The 150-day cumulative incidence of PGF and leukopenia after COVID-19 between short and long-term infection group. (**A**) PGF; (**B**) leukopenia. *PGF* poor graft function.

The 150-day cumulative incidence of PGF after COVID-19 was 11.8% (95% CI 6.4–17.2%) versus 23.3% (95% CI 10.5–36.1%) (*P* = 0.071) between non-serious and serious infection group, which was 9.0% (95% CI 3.0–15.0%) versus 20.0% (95% CI 11.7–28.3%) (*P* = 0.045) between short- and long-term infection group (Fig. 1A).

The 150-day cumulative incidence of leukopenia after COVID-19 was 19.9% (95% CI 13.2–26.6%) versus 14.0% (95% CI 3.5–24.5%) (*P* = 0.325) between non-serious and serious infection group, which was 12.4% (95% CI 5.5–19.3%) versus 24.4% (95% CI 15.5–33.3%) (*P* = 0.049) between short- and long-term infection group (Fig. 1B).

The 150-day cumulative incidence of thrombocytopenia after COVID-19 was 5.1% (95% CI 1.4–8.8%) versus 9.3% (95% CI 0.5–18.1%) (*P* = 0.331) between non-serious and serious infection group, which was 5.6% (95% CI 0.8–10.4%) versus 6.7% (95% CI 1.5–11.9%) between short- and long-term infection group (*P* = 0.779).

The median duration of leukopenia, thrombocytopenia, and PGF was 14 days (range 4–118) days, 10 days (range 8–78) days, and 17 days (range 9–78) days, respectively. Until the last follow up, leukopenia, thrombocytopenia and PGF were still persistence 22 patients.

In multivariable analysis, after adjusted by other factors, the duration of COVID-19 was associated with PGF (hazard ratio [HR], 2.31; 95% CI 1.04–5.11; *P* = 0.039) and leukopenia (HR 2.29; 95% CI 1.04–5.07; *P* = 0.04) (Table 2). The other risk factors for PGF and leukopenia were showed in Supplementary Table 4.

**Table 2:** Multivariate analysis of risk factors for the 150-day clinical outcomes after COVID-19 infection.

Outcomes	HR (95% CI)	*P* value
PGF after COVID-19		
The duration of COVID-19		0.039
Short-term	1	
Long-term	2.31 (1.04–5.11)	
Leukopenia after COVID-19		
The duration of COVID-19		0.040
Short-term	1	
Long-term	2.29 (1.04–5.07)	
CMV disease after COVID-19		
The severity of COVID-19		0.005
Non-serious	1	
Serious	20.15 (2.43–167.36)	
NRM after COVID-19		
The severity of COVID-19		< 0.0001
Non-serious	1	
Serious	17.26 (4.87–61.21)	
OS after COVID-19		
The severity of COVID-19		< 0.0001
Non-serious	1	
Serious	14.00 (5.87–33.42)	

*PGF* poor graft function, *CMV* cytomegalovirus, *NRM* non-relapse mortality, *OS* overall survival.

### The severity and duration of COVID-19 and aGVHD

A total of 5 patients showed aGVHD after COVID 19 infection, and the cumulative incidence of total aGVHD was 2.8% (95% CI 0.4–5.2%) after COVID-19. The cumulative incidence of aGVHD after COVID-19 was 3.7% (95% CI 0.5–6.9%) versus 0% (*P* = 0.204), respectively, between non-serious and serious infection group, which was 3.4% (95% CI 0.4–7.2%) versus 2.2% (95% CI 0.9–5.3%) (*P* = 0.651), respectively, between short- and long-term infection group. No risk factors were associated with aGVHD in multivariable analysis.

### The severity and duration of COVID-19 and chronic GVHD (cGVHD)

A total of 4, 4 and 3 patients showed mild, moderate, and severe cGVHD after COVID 19 infection, and the cumulative incidence of cGVHD was 6.70% after COVID-19. The 150-day cumulative incidence of cGVHD after COVID-19 was 7.4% (95% CI 2.9–11.8%) versus 4.7% (95% CI − 1.7–11.1%), respectively, between non-serious and serious infection group (*P* = 0.544). The 150-day cumulative incidence of cGVHD after COVID-19 was 6.7% (95% CI 1.5–11.9%) versus 5.6% (95% CI 0.8–10.4%), respectively, between short- and long-term infection group (*P* = 0.741). No risk factors were associated with cGVHD in multivariable analysis.

### The severity and duration of COVID-19 and other infection

**Figure 2:**
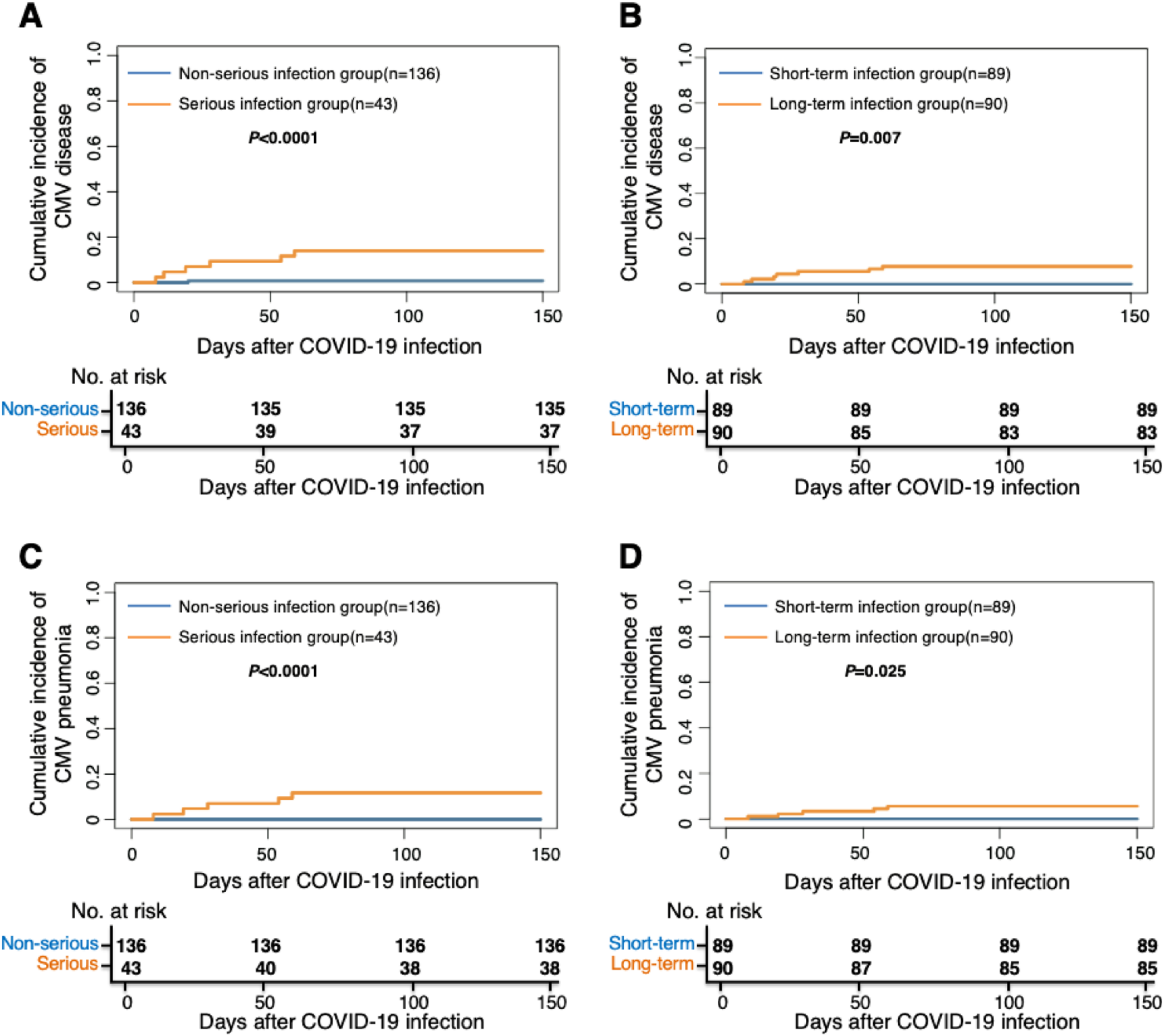
The association between COVID-19 and CMV disease. The 150-day cumulative incidence of CMV disease after COVID-19 between (**A**) non-serious and serious infection group; (**B**) short and long-term infection group. The 150-day cumulative incidence of CMV pneumonia after COVID-19 between (**C**) non-serious and serious infection group; (**D**) short and long-term infection group. *CMV* cytomegalovirus.

A total of 34 and 7 patients showed CMV DNAemia and CMV disease (CMV pneumonia: 5, CMV gastrointestinal disease: 1, CMV encephalitis + retinitis: 1) after COVID 19 infection. The 150-day cumulative incidence of CMV DNAemia after COVID-19 was 19.9% (95% CI 13.2–26.6%) versus 11.6% (95% CI 1.9–21.3%) (*P* = 0.204), respectively, between non-serious and serious infection group, which was 14.6% (95% CI 7.2–22.0%) versus 23.3% (95% CI 14.5–32.1%) (*P* = 0.118), respectively, between short- and long-term infection group. The 150-day cumulative incidence of CMV disease after COVID-19 was 0.7% (95% CI 0.7–2.1%) versus 14.0% (95% CI 3.5–24.5%) (*P* < 0.0001, Fig. 2A), respectively, between non-serious and serious infection group, which was 0% versus 7.8% (95% CI 2.2–13.4%) (*P* = 0.007, Fig. 2B), respectively, between short- and long-term infection group. Particularly, the 150-day cumulative incidence of CMV pneumonia after COVID-19 was 0% versus 11.6% (95% CI 1.9–21.4%) (*P* < 0.0001, Fig. 2C), respectively, between non-serious and serious infection group, which was 0% versus 5.6% (95% CI 0.8–10.4%) (*P* = 0.0245, Fig. 2D), respectively, between short- and long-term infection group. In multivariable analysis, after adjusted by other factors, the severity of COVID-19 was associated with CMV disease (HR, 20.15; 95% CI 2.43–167.36, *P* = 0.005) (Table 2).

A total of 11 and 3 patients showed Epstein-Barr virus (EBV) DNAemia and EBV associated posttransplant lymphoproliferative disorders (PTLD) after COVID-19. The 150-day cumulative incidence of EBV DNAemia after COVID-19 was 7.4% (95% CI 3.0–11.8%) versus 0.00% (*P* = 0.068), respectively, between non-serious and serious infection group. The 150-day cumulative incidence of EBV DNAemia after COVID-19 was 5.6% (95% CI 0.8–10.4%) versus 5.6% (95% CI 0.8–10.4%) (*P* = 0.968), respectively, between short- and long-term infection group. All the 3 PTLD patients were in the non-serious group. No risk factors were associated with EBV DNAemia and PTLD in multivariable analysis.

### The severity and duration of COVID-19 and mortality and survival

A total of 27 patients died after COVID-19, and the caused were summarized in Table 3. The most common cause was infection besides of COVID-19 (*n* = 9, 33.3%), followed by relapse (*n* = 7, 25.9%) and COVID-19 (*n* = 4, 14.8%).

**Table 3:** Cause of death.

Cause of death	n (%)
Infection besides of COVID-19	9 (33.3)
CMV pneumonia	2 (7.4)
CMV gastrointestinal disease	1 (3.7)
Klebsiella pneumoniae sepsis	1 (3.7)
Severe pneumonia of unknown etiology	5 (18.5)
Relapse	7 (25.9)
COVID-19	4 (14.8)
Cerebrovascular disease	4 (14.8)
PGF	1 (3.7)
aGVHD	1 (3.7)
Arrhythmia	1 (3.7)

*PGF* poor graft function, *CMV* cytomegalovirus, *aGVHD* acute graft versus disease.

**Figure 3:**
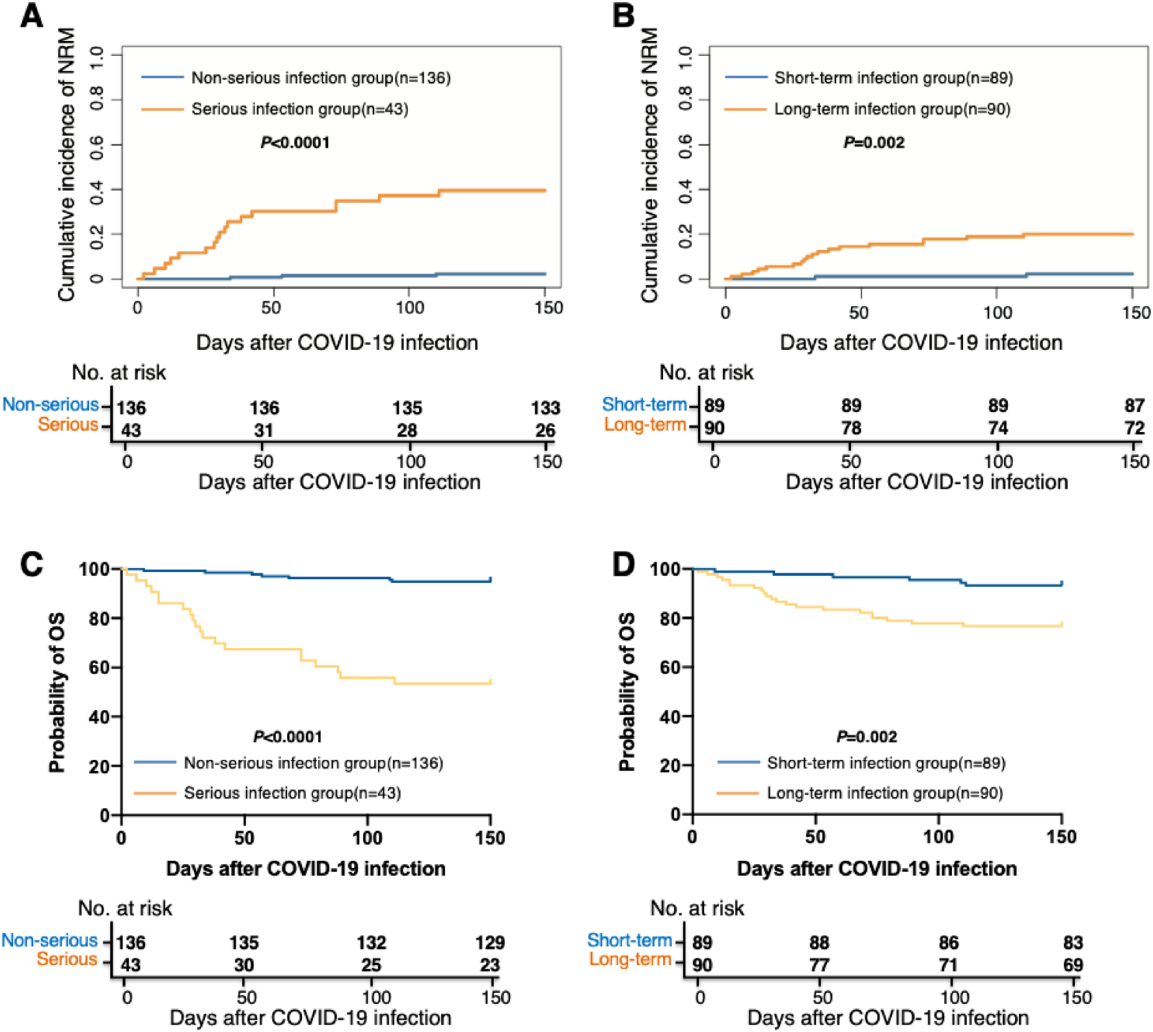
The association between COVID-19 and survival. The 150-day cumulative incidence of NRM after COVID-19 between (**A**) non-serious and serious infection group; (**B**) short and long-term infection group. The 150-day probability of OS after COVID-19 infection between (**C**) non-serious and serious infection group; (**D**) short and long-term infection group. *NRM* non-relapse mortality, *OS* overall survival.

The 150-day cumulative incidence of non-relapse mortality (NRM) after COVID-19 infection was 11.2% (95% CI 6.6–15.8%), which was 2.2% (95% CI 0.3–4.7%) and 39.5% (95% CI 24.6–54.4%) between non-serious and serious infection group (*P* < 0.0001, Fig. 3A), and was 2.2% (95% CI 0.9–5.3%) and 20.0% (95% CI 11.7–28.3%) (*P* = 0.002, Fig. 3B) between short- and long-term infection group.

The 150-day probability of overall survival (OS) after COVID-19 infection was 84.9% (95% CI 79.6–90.2%), which was 94.9% (95% CI 91.2–98.6%) and 53.5% (95% CI 38.1–68.5%) between non-serious and serious infection group (*P* < 0.0001, Fig. 3C), and was 93.3% (95% CI 88.1–98.5%) and 76.7% (95% CI 67.9–85.5%) (*P* = 0.002, Fig. 3D) between short- and long-term infection group.

In multivariable analysis, after adjusted by other factors, the severity of COVID-19 was associated with NRM (HR, 17.26; 95% CI 4.87–61.21, *P* < 0.0001). The severity of COVID-19 were associated with OS (HR, 14.00; 95% CI 5.87–33.42, *P* < 0.0001) (Table 2), The other risk factors for NRM and OS were showed in Supplementary Table 4.

### Clinical outcomes of patients with and without COVID 19 infection

**Figure 4:**
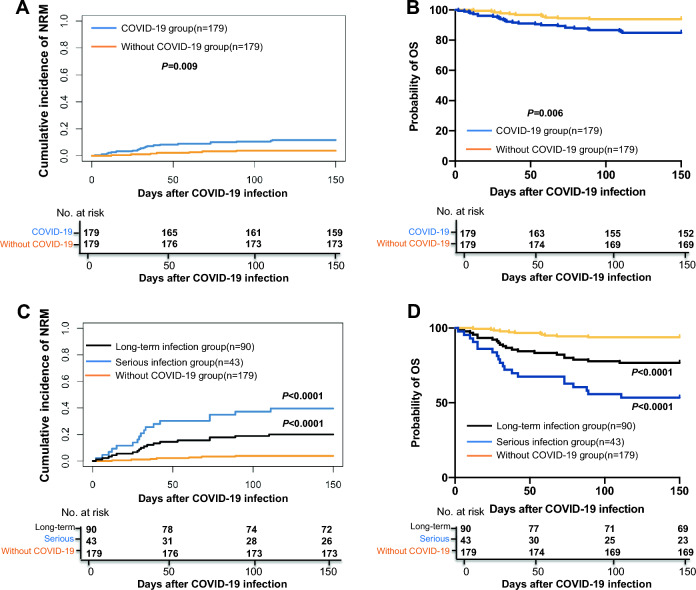
Clinical outcomes of patients with and without COVID 19 infection. (**A**) The 150-day cumulative incidence of NRM in the group with and without COVID-19 infection; (**B**) The 150-day cumulative incidence of OS in the group with and without COVID-19 infection; (**C**) The 150-day cumulative incidence of NRM in the group without COVID-19 infection, serious infection and long-term infection; (**D**) The 150-day cumulative incidence of OS in the group without COVID-19 infection, serious infection and long-term infection, *NRM* non-relapse mortality, *OS* overall survival.

A total of 179 patients without COVID-19 infection were enrolled as controlled and the characteristics between patients with and without COVID-19 were showed in Supplementary Table 5. The 150-day probability of NRM and OS were 11.2% (95% CI 6.6–15.8%) versus 3.9% (95% CI 1.1–6.7%) with *P* = 0.009 and 84.9% (95% CI 79.6–90.2%) versus 93.9% (95% CI: 88.7%–99.1%) with *P* = 0.006, respectively, for patients in the group with and without COVID-19 infection (Fig. 4A,B). The probability of NRM and OS for patients without COVID-19 infections were superior to those in serious infection group or long-term infection group (Fig. 4C,D).

## Discussion

In the present study enrolled consecutive hospitalized COVID-19 patients, 24% of them showed serious infection, and more than half of the patients experienced long-term COVID-19 infection (i.e., more than 18 days). The 150-day cumulative incidence of PGF, leukopenia, CMV disease, CMV pneumonia, NRM, and OS after COVID-19 was significantly higher in long-term infection group compared with short-term infection group. In addition, the 150-day cumulative incidence of CMV disease, CMV pneumonia, NRM, and OS after COVID-19 was also significantly higher in serious infection group compared with non-serious infection group. Thus far, this is the first study identify the influence of severity and duration of SARS-CoV-2 infection on complications after allo-HSCT in the Omicron era.

Viral infection is one of the most important causes of PGF. Parvovirus B19 was the most common virus which can lead to rejection and PGF^24,25^. In addition, herpes virus could also induce PGF. For example, the association of HHV-6 infections and thrombocytopenia has been reported since 1990s^26^, and several studies observed the association between HHV-6 infections and delayed platelet engraftment in allo-HSCT recipients^27,28^. Some studies also reported that CMV infection was associated with thrombocytopenia^29^ and PGF^16^. The potential mechanism including impairment of virus on hematopoietic microenvironment or hematologic toxicities of anti-virus drugs. Herpes simplex virus and CMV can infect hematopoietic progenitor cells^30–32^, and the treatments of CMV such as gancilovir and forscarnet could lead to myelosuppression. Similarly, several studies also reported SARS-CoV-2 infection could cause anemia^33–35^, thrombocytopenia^34–36^, and aplastic anemia^37^. Thus, it is suggested that we should pay attention to PGF in patients with SARS-CoV-2 infection, particularly for those with long-term infection.

In addition, we observed that the incidence of CMV disease, particularly the CMV pneumonia, significantly increased in those with serious and/or long-term SARS-CoV-2 infection. Some data suggested that some viruses, such as HHV-6 and HHV-7, could take part in CMV reactivation or progression in immunosuppressed patients^38,39^. Lymphopenia is the hallmark of severe COVID-19 presentation, and previous studies observed that both the numbers of circulating T, B, and NK cells and antiviral cytokine production capability decreased in patients with COVID-19, particularly in those required intensive care^40–42^. Drylewicz et al.^43^ show that reconstitution of CD4^+^ T cells and NK cells after allo-HSCT is important for CMV prophylaxis. Thus, we speculated that COVID-19 infection may increase the susceptibility of CMV reactivation after allo-HSCT, which should be further confirmed in the future.

Previous studies have shown that the mortality rate range 12.5–22% in allo-HSCT recipients after COVID-19 infection^44–46^. While in the immunodeficient patients infected with Omicron variant, the mortality rate was 10.5% in the advanced malignancy cohort^23^. Our data suggested the 150-day cumulative incidence of NRM after COVID-19 infection was 11.2% in allo-HSCT recipients, which was similar to the previous studies. In addition, we only enrolled the hospitalized COVID-19 patients, which may also contribute to the relatively high NRM.

As mentioned above, long-term SARS-CoV-2 infection increased the risk of PGF, CMV disease, and NRM, this suggested that how to clear the SARS-CoV-2 timely is critical to improve the outcomes of these patients. Besides of the anti-viral drug (e.g., Nirmatrelvir/Ritonavir), the intense of immunosuppressive therapy could influence the viral clearance and was associated with severity and persistence of infection. Thus, allo-HSCT recipients may taper the immunosuppressive therapy after SARS-CoV-2 infection.

There were some limitations in the present study. This was a single center, observational cohort study and the followed-up was still relatively short, so we should further identify the long-term impact of Omicron variant infection on post-transplant complications in future.

In summary, we observed that the severity and duration of COVID-19 infection were associated with PGF, leukopenia, CMV disease, CMV pneumonia, NRM, and survival after allo-HSCT. Thus, how to prevent and clear the SARS-CoV-2 infection effectively is still critical for allo-HSCT recipients in the Omicron era.

## Materials and methods

### Patients

We performed an observational prospective study in Peking University, Institute of Hematology (PUIH, *n* = 179). Including criteria were as followed: 1) the hospitalized patients had received an allo-HSCT in PUIH at any time before the diagnosis of COVID-19, and 2) have laboratory-confirmed (polymerase chain reaction or antigenic test) COVID-19. In order to compare the clinical outcome between patients with and without COVID-19, a historical cohort included patients receiving allo-HSCT from August 1 2016 to December 1 2022 were enrolled and they were matched for age, sex, underlying disease, allo-HSCT time and time after allo-HSCT in a 1: 1 ratio. The study was approved by the Ethics Committee of Peking University People’s Hospital, and written informed consent was obtained from all subjects before study entry, in accordance with the Declaration of Helsinki.

### Transplantation protocols

Donor selection, human leukocyte antigen typing, graft harvesting, conditioning regimen, GVHD, and infection prophylaxis were performed as previously described^1^. Comorbidities in HSCT recipients were assessed according to the hematopoietic cell transplantation–specific comorbidity index (HCT-CI).

### Clinical definitions and assessments

During our study period, the Omicron variant was predominant in Beijing, China. The severity of COVID-19 was categorized as asymptomatic infection, mild, moderate, severe, and critical illness according to guidelines of National Institutes of Health (Supplementary methods). Severe illness and critical illness were classified as serious infection, and asymptomatic infection, mild illness and moderate illness were classified as non-serious infection in the present study. The duration of COVID-19 infection was defined as the time of the patients with persistent signs and symptoms with polymerase chain reaction or antigenic test positivity.

PGF was defined as the presence of 2 or 3 cytopenic counts (ANC ≤ 0.5 × 10^9^/L, platelet ≤ 20 × 10^9^/L, or hemoglobin ≤ 70 g/L) for at least 3 consecutive days beyond day 28 post-transplantation or dependence on transfusion and granulocyte colony-stimulating factor (G-CSF), in the presence of complete donor chimerism. Leukopenia was defined as ANC ≤ 0.5 × 10^9^/L alone for at least 3 consecutive days beyond day 28 post-transplantation or dependence on G-CSF, thrombocytopenia was defined as engraftment of peripheral blood cell lines (ANC > 0.5 × 10^9^/L and hemoglobin > 70 g/L without transfusion support) other than a platelet count < 20 × 10^9^/L or dependence on platelet transfusions for at least 7 consecutive days, in the presence of complete donor chimerism. Patients with evidence of hematological relapse post-HSCT were excluded. GVHD was diagnosed and graded based on international criteria^47^. CMV infection was diagnosed according to the definition of CMV Drug Development Forum^48^. PTLD was diagnosed according to the Sixth European Conference on Infections in Leukemia guidelines^49^.

Relapse was defined by morphologic evidence of disease in peripheral blood, bone marrow, or extramedullary site samples or by the recurrence and sustained presence of pre-transplantation chromosomal abnormalities. NRM was defined as death without disease progression or relapse. OS was defined as the time from transplantation to death from any cause.

### Statistical analysis

Characteristics of patients were summarized by descriptive statistics, that is, using counts and percentages for categorical variables and using median and range for continuous variables. Subject variables were compared using the χ^2^ test for categorical variables and the Mann–Whitney *U* test for continuous variables. Multivariate analyses were performed using the Cox proportional hazards model for survival to identify the independent prognostic variables (Supplementary methods). The parameters with *P* < 0.10 according to the univariate analysis were entered into a multivariate model (Supplementary Table 3). Cumulative incidence curves were used in a competing risk setting, with relapse treated as a competing event, to calculate NRM probabilities, and with death and relapse as the competing risks for PGF, infection, and GVHD. The probability of survival was estimated with the Kaplan–Meier method and were compared using the log-rank test. Statistical analyses were performed using 1-way analysis of variance (ANOVA) for comparisons among the groups. Analyses were performed using GraphPad Prism 6.0 (GraphPad Software), SPSS 24 (SPSS Inc./IBM, Armonk, NY, USA) and R version 3.4.4 (The R Foundation for Statistical Computing). Unless otherwise specified, all *P* values were 2-sided and *P* < 0.05 was considered significant.

### Supplementary Information

Supplementary Information.

## Data Availability

The datasets used and/or analysed during the current study available from the corresponding author on reasonable request.

## References

[1] Xu LP (2018). The consensus on indications, conditioning regimen, and donor selection of allogeneic hematopoietic cell transplantation for hematological diseases in China-recommendations from the Chinese Society of Hematology. J. Hematol. Oncol..

[2] Lv M, Shen MZ, Mo XD (2023). Development of allogeneic hematopoietic stem cell transplantation in 2022: Regenerating "Groot" to heal the world. Innovation.

[3] Arnaout K (2014). Complications of allogeneic hematopoietic stem cell transplantation. Cancer Invest..

[4] Pei XY (2022). Comparable anti-CMV responses of transplant donor and third-party CMV-specific T cells for treatment of CMV infection after allogeneic stem cell transplantation. Cell. Mol. Immunol..

[5] Chakraborty I, Maity P (2020). COVID-19 outbreak: Migration, effects on society, global environment and prevention. Sci. Total Environ..

[6] Liu Y, Rocklöv J (2022). The effective reproductive number of the Omicron variant of SARS-CoV-2 is several times relative to Delta. J. Travel Med..

[7] Busca A (2023). Outcome of COVID-19 in allogeneic stem cell transplant recipients: Results from the EPICOVIDEHA registry. Front. Immunol..

[8] Shahzad M (2022). Impact of COVID-19 in hematopoietic stem cell transplant recipients: A systematic review and meta-analysis. Transpl. Infect. Dis..

[9] Hui KPY (2022). Replication of SARS-CoV-2 Omicron BA.2 variant in ex vivo cultures of the human upper and lower respiratory tract. EBioMedicine.

[10] Hui KPY (2022). SARS-CoV-2 Omicron variant replication in human bronchus and lung ex vivo. Nature.

[11] Li H (2022). The effects of vaccination on the disease severity and factors for viral clearance and hospitalization in Omicron-infected patients: A retrospective observational cohort study from recent regional outbreaks in China. Front. Cell. Infect. Microbiol..

[12] Zeng QL (2022). Clinical characteristics of omicron SARS-CoV-2 variant infection after non-mRNA-based vaccination in China. Front. Microbiol..

[13] Christensen PA (2022). Signals of significantly increased vaccine breakthrough, decreased hospitalization rates, and less severe disease in patients with coronavirus disease 2019 caused by the omicron variant of severe acute respiratory syndrome coronavirus 2 in Houston, Texas. Am. J. Pathol..

[14] Smith DJ (2022). COVID-19 mortality and vaccine coverage: Hong Kong Special Administrative Region, China, January 6, 2022-March 21, 2022. MMWR.

[15] Mo XD (2016). Late-onset severe pneumonia after allogeneic hematopoietic stem cell transplantation: prognostic factors and treatments. Transpl. Infect. Dis..

[16] Sun YQ (2019). Virus reactivation and low dose of CD34+ cell, rather than haploidentical transplantation, were associated with secondary poor graft function within the first 100 days after allogeneic stem cell transplantation. Ann. Hematol..

[17] Gratama JW (1987). Herpes-virus immunity and acute graft-versus-host disease. Lancet.

[18] Rashidi A, Said B, Ebadi M, Weisdorf DJ (2018). Human herpesvirus-6 reactivation and acute graft-versus-host disease. Biol. Blood Marrow Transplant..

[19] Akahoshi Y (2021). Effect of cytomegalovirus reactivation with or without acute graft-versus-host disease on the risk of nonrelapse mortality. Clin. Infect. Dis..

[20] Altuntas F (2021). COVID-19 in hematopoietic cell transplant recipients. Bone Marrow Transplant..

[21] Barhoom D (2021). Clinical effects of COVID-19 on hematopoietic stem cell transplant outcomes in pediatric patients. Exp. Clin. Transplant..

[22] Lupo-Stanghellini MT (2021). COVID-19 in recipients of allogeneic stem cell transplantation: favorable outcome. Bone Marrow Transplant..

[23] Zhu XL (2023). COVID-19 infection in patients with haematological malignancies: A single-centre survey in the latest Omicron wave in China. Br. J. Haematol..

[24] Wasak-Szulkowska E, Grabarczyk P, Rzepecki P (2008). Pure red cell aplasia due to parvovirus B19 infection transmitted probably through hematopoietic stem cell transplantation. Transpl. Infect. Dis..

[25] Eid AJ, Brown RA, Patel R, Razonable RR (2006). Parvovirus B19 infection after transplantation: a review of 98 cases. Clin. Infect. Dis..

[26] Carrigan DR, Knox KK (1994). Human herpesvirus 6 (HHV-6) isolation from bone marrow: HHV-6-associated bone marrow suppression in bone marrow transplant patients. Blood.

[27] Ljungman P (2000). High levels of human herpesvirus 6 DNA in peripheral blood leucocytes are correlated to platelet engraftment and disease in allogeneic stem cell transplant patients. Br. J. Haematol..

[28] Zerr DM (2005). Clinical outcomes of human herpesvirus 6 reactivation after hematopoietic stem cell transplantation. Clin. Infect. Dis..

[29] Dominietto A (2001). Factors influencing haematological recovery after allogeneic haemopoietic stem cell transplants: graft-versus-host disease, donor type, cytomegalovirus infections and cell dose. Br. J. Haematol..

[30] Humby MS, O'Connor CM (2015). Human cytomegalovirus US28 is important for latent infection of hematopoietic progenitor cells. J. Virol..

[31] Isomura H (1997). Suppressive effects of human herpesvirus 6 on in vitro colony formation of hematopoietic progenitor cells. J. Med. Virol..

[32] Maciejewski JP, St Jeor SC (1999). Human cytomegalovirus infection of human hematopoietic progenitor cells. Leuk Lymphoma.

[33] Al-Kuraishy HM, Al-Gareeb AI, Kaushik A, Kujawska M, Batiha GE (2022). Hemolytic anemia in COVID-19. Ann. Hematol..

[34] Elahi S (2022). Hematopoietic responses to SARS-CoV-2 infection. Cell. Mol. Life Sci..

[35] Rahi MS (2021). Hematologic disorders associated with COVID-19: a review. Ann. Hematol..

[36] Mei H, Luo L, Hu Y (2020). Thrombocytopenia and thrombosis in hospitalized patients with COVID-19. J. Hematol. Oncol..

[37] Avenoso D (2022). SARS-CoV-2 infection in aplastic anemia. Haematologica.

[38] Quintela A (2016). HHV-6 infection after allogeneic hematopoietic stem cell transplantation: From chromosomal integration to viral co-infections and T-cell reconstitution patterns. J. Infect..

[39] Dzieciatkowski T (2016). Analysis of the shedding of three β-herpesviruses DNA in Polish patients subjected to allogeneic hematopoietic stem cell transplantation: Six-year follow up. J. Clin. Virol..

[40] Mazzoni A (2020). Impaired immune cell cytotoxicity in severe COVID-19 is IL-6 dependent. J. Clin. Invest..

[41] Chen G (2020). Clinical and immunological features of severe and moderate coronavirus disease 2019. J. Clin. Invest..

[42] Qin C (2020). Dysregulation of immune response in patients with coronavirus 2019 (COVID-19) in Wuhan, China. Clin. Infect. Dis..

[43] Drylewicz J (2016). Rapid reconstitution of CD4 T cells and NK cells protects against CMV-reactivation after allogeneic stem cell transplantation. J. Transl. Med..

[44] Sharma A (2021). Clinical characteristics and outcomes of COVID-19 in haematopoietic stem-cell transplantation recipients: an observational cohort study. Lancet Haematol..

[45] Varma A (2020). COVID-19 infection in hematopoietic cell transplantation: age, time from transplant and steroids matter. Leukemia.

[46] Vicent MG (2020). COVID-19 in pediatric hematopoietic stem cell transplantation: The experience of Spanish Group of Transplant (GETMON/GETH). Pediatr. Blood Cancer.

[47] Schoemans HM (2018). EBMT-NIH-CIBMTR Task Force position statement on standardized terminology & guidance for graft-versus-host disease assessment. Bone Marrow Transplant..

[48] Ljungman P (2017). Definitions of cytomegalovirus infection and disease in transplant patients for use in clinical trials. Clin. Infect. Dis..

[49] Styczynski J (2016). Management of Epstein-Barr Virus infections and post-transplant lymphoproliferative disorders in patients after allogeneic hematopoietic stem cell transplantation: Sixth European Conference on Infections in Leukemia (ECIL-6) guidelines. Haematologica.

