# Supplementary material for: COVID-19 was associated with the complications after allogeneic hematopoietic stem cell transplantation
Source: Sci Rep. 2024 May 23;14:11778. doi: 10.1038/s41598-024-62731-7 (PMC11116404; doi:10.1038/s41598-024-62731-7)
Supplement: Supplementary file 1 — Supplementary Information. [file 41598_2024_62731_MOESM1_ESM.docx]

**Title: COVID-19 was associated with the complications after allogeneic hematopoietic stem cell transplantation**

**Shorting running title: COVID19 and post-HSCT complications**

**Authors:** Qi Wen^1^**^†^**, Ze Guo^1^**^†^**, Xiao-Hui Zhang^1^, Lan-Ping Xu^1^, Yu Wang^1^, Chen-Hua Yan^1^, Huan Chen^1^, Yu-Hong Chen^1^, Wei Han^1^, Feng-Rong Wang^1^, Yu-Qian Sun^1^, Xiao-Jun Huang^1,2^, and Xiao-Dong Mo^1,2*^

**Affiliations:**

^1^Peking University People's Hospital, Peking University Institute of Hematology, National Clinical Research Center for Hematologic Disease, Beijing Key Laboratory of Hematopoietic Stem Cell Transplantation, Beijing, China;

^2^Research Unit of Key Technique for Diagnosis and Treatments of Hematologic Malignancies, Chinese Academy of Medical Sciences, Beijing 2019RU029, China.

Qi Wen and Ze Guo contributed equally to this manuscript.

*Correspondence: Prof. Xiao-Dong Mo; Peking University People’s Hospital, Peking University Institute of Hematology, No. 11 Xizhimen South Street, Xicheng District, Beijing 100044, China; E-mail: moxiaodong@pkuph.edu.cn; Tel: 8610-8832-4577

**Supplementary Methods**

**Clinical definitions and assessments of COVID-19**

During our study period, the Omicron variant was predominant in Beijing, China. The severity of COVID-19 was categorized as asymptomatic infection, mild, moderate, severe, and critical illness according to guidelines of National Institutes of Health as followed:
1) Asymptomatic infection: Patients who test positive for SARS-CoV-2 using a virologic test but who have no symptoms that are consistent with COVID-19;
2) Mild illness: Patients who have any of the various signs and symptoms of COVID-19, without shortness of breath, dyspnea, or abnormal chest imaging;
3) Moderate illness: Patients have evidence of lower respiratory disease during clinical assessment or imaging and have an oxygen saturation measured by pulse oximetry (SpO2) ≥ 94% on room air at sea level;
4) Severe illness: Patients have SpO2 <94% on room air at sea level, a ratio of arterial partial pressure of oxygen to fraction of inspired oxygen < 300 mmHg, a respiratory rate > 30 breaths/min, or lung infiltrates >50%;
5) Critical illness: Individuals who have respiratory failure, septic shock, and/or multiple organ dysfunction.

Multivariate analysis

Hazard ratios (HRs) for ORR, mortality, and survival were estimated in a multivariate analysis using Cox proportional hazards regression. Independent variables with *P* > 0.1 were sequentially excluded from the model, and *P* < 0.05 was considered to be statistically significant. These following variables were included: the severity of COVID-19 (non-serious vs serious COVID-19 infection), the duration of COVID-19 (≤ 18 days vs > 18 days), gender, patient age (using the median value as the cutoff point, < 40 years vs ≥ 40 years), HCT-CI before HSCT (< 3 scores vs ≥ 3 scores), donor-recipients gender matched (female to male vs others), donor type (identical sibling donor vs alternative donor), donor-recipient blood group matched (mismatched ys matched), mononuclear and CD34^+^ cell counts in graft (using the median value as the cutoff point), and lymphocytes at COVID-19 diagnosis (using the median value as the cutoff point).

**Supplementary table 1. Characteristics between serious and non-serious COVID-19**

| **Characteristics** | **Serious (*n* = 43)** | **Non-serious (*n* = 136)** |
| --- | --- | --- |
| Gender, male/female, n (%) | 25 (58.1)/18 (41.9) | 83 (61.0)/53 (39.0) |
| Age(years), range | 44 (8-75) | 37 (5-67) |
| Underlying disease, n (%) |  |  |
| AL | 30 (69.8) | 101 (74.3) |
| MDS | 7 (16.3) | 17 (12.5) |
| AA | 0 (0.0) | 5 (3.7) |
| Lymphoma | 0 (0.0) | 10 (7.4) |
| Others | 5 (11.6) | 4 (2.9) |
| Donor match, n (%) |  |  |
| HLA-matched sibling donor | 6 (14.0) | 25 (18.4) |
| HLA-matched unrelated donor | 1 (2.3) | 5 (3.7) |
| Haploidentical related donor | 36 (83.7) | 106 (77.9) |
| Blood group matched, n (%) |  |  |
| Matched | 22 (51.2) | 81 (59.6) |
| Minor mismatched | 8 (18.6) | 22 (16.2) |
| Major mismatched | 13 (30.2) | 33 (24.3) |
| HCT-CI before HSCT, n (%) |  |  |
| 0 (low risk) | 29 (67.4) | 94 (69.1) |
| 1-2 (intermediate risk) | 10 (23.3) | 31 (22.8) |
| ≥3 (high risk) | 4 (9.3) | 11 (8.1) |
| Median counts of MNC in graft, range (×10^8^/kg) | 9.51 (5.43-14.25) | 9.53 (3.83-19.90) |
| Median counts of CD34^+^ cell in graft, range (×10^6^/kg) | 2.88 (1.18-10.28) | 3.17 (0.48-17.20) |
| Conditioning, n (%) |  |  |
| Chemotherapy-based | 40 (93.0) | 128 (94.1) |
| TBI-based | 3 (7.0) | 8 (5.9) |
| Median counts of lymphocytes at COVID-19 diagnosis, range (×10^9^/L) | 1.29 (0.10-3.83) | 1.00 (0.00-6.21) |
| Duration of COVID-19 (days), range ** | 30 (3–102) | 14 (2-71) |

**Abbreviations:** AL, acute leukemia; MDS, myelodysplastic syndromes; AA, aplastic anemia; HLA, human leukocyte antigen; HCT-CI, Hematopoietic Cell Transplantation–Specific Comorbidity Index; MNC, mononuclear cell; TBI, total body irradiation.

*P* < 0.05, *; *P* < 0.01, **.

**Supplementary table 2.** **Characteristics between short-term and long-term COVID-19**

| **Characteristics** | **Short-term (*n* = 89)** | **Long-term (*n* = 90)** |
| --- | --- | --- |
| Gender, male/female, n (%) | 50 (56.2)/39 (43.8) | 58 (16.8)/32 (16.8) |
| Age(years), range | 37 (5-75) | 41.5 (8-67) |
| Underlying disease, n (%) |  |  |
| AL | 68 (76.4) | 63 (70.0) |
| MDS | 11 (12.4) | 13 (14.4) |
| AA | 2 (2.2) | 3 (3.3) |
| Lymphoma | 5 (5.6) | 5 (5.6) |
| Others | 3 (3.4) | 6 (6.7) |
| Donor match, n (%) |  |  |
| HLA-matched sibling donor | 19 (21.3) | 12 (13.3) |
| HLA-matched unrelated donor | 4 (4.5) | 2 (2.2) |
| Haploidentical related donor | 66 (74.2) | 76 (84.4) |
| Blood group matched, n (%) |  |  |
| Matched | 51 (57.3) | 52 (57.8) |
| Minor mismatched | 14 (15.7) | 16 (17.8) |
| Major mismatched | 24 (27.0) | 22 (24.4) |
| HCT-CI before HSCT, n (%) |  |  |
| 0 (low risk) | 65 (73.0) | 58 (64.4) |
| 1-2 (intermediate risk) | 18 (20.2) | 23 (25.6) |
| ≥3 (high risk) | 6 (6.7) | 9 (10.0) |
| Median counts of MNC in graft, range (× 10^8^/kg) | 9.56 (5.77-18.93) | 9.47 (3.83-19.90) |
| Median counts of CD34^+^ cell in graft, range (× 10^6^/kg) | 2.99 (0.95-17.20) | 3.12 (0.48-16.09) |
| Conditioning, n (%) |  |  |
| Chemotherapy-based | 86 (96.6) | 82 (91.1) |
| TBI-based | 3 (3.4) | 8 (8.9) |
| Median counts of lymphocytes at COVID-19 diagnosis, range (×10^9^/L) | 1.00 (0.00-5.46) | 1.03 (0.00-6.21) |
| COVID-19, n (%) |  |  |
| Asymptomatic infection | 8 (9.0) | 6 (6.7) |
| Mild illness | 46 (51.7) | 32 (35.6) |
| Moderate illness | 25 (28.1) | 19 (21.1) |
| Severe illness | 8 (9.0) | 14 (15.6) |
| Critical illness ** | 2 (2.2) | 19 (21.1) |

**Abbreviations:** AL, acute leukemia; MDS, myelodysplastic syndromes; AA, aplastic anemia; HLA, human leukocyte antigen; HCT-CI, Hematopoietic Cell Transplantation–Specific Comorbidity Index; MNC, mononuclear cell; TBI, total body irradiation.

*P* < 0.05, *; *P* < 0.01, **.

**Supplementary table 3. Univariate analysis of risk factors for the 150-day clinical outcomes after COVID-19 infection**

| **Outcomes** | **HR (95% CI)** | ***P* value** |
| --- | --- | --- |
| **PGF after COVID-19** |  |  |
| The duration of COVID-19 | 2.29 (0.99-5.26) | 0.052 |
| The severity of COVID-19 | 2.05 (0.93-4.51) | 0.075 |
| Age | 1.37 (0.63-2.98) | 0.429 |
| Gender | 1.30 (0.60-2.82) | 0.499 |
| Donor-recipients gender matched | 0.81 (0.32-2.01) | 0.646 |
| Donor type | 0.60 (0.18-2.01) | 0.410 |
| Donor-recipient blood group matched | 1.68 (0.78-3.63) | 0.187 |
| HCT-CI before HSCT | 0.40 (0.06-2.98) | 0.374 |
| MNC counts in graft | 0.59 (0.27-1.30) | 0.193 |
| CD34^+^ cell counts in graft | 1.37 (0.63-2.98) | 0.432 |
| Lymphocytes at COVID-19 diagnosis | 0.34 (0.14-0.81) | 0.015 |
| Time from allo-HSCT to COVID-19 | 0.88 (0.72–1.08) | 0.196 |
| **Leukopenia after COVID-19** |  |  |
| The duration of COVID-19 | 2.04 (0.99-4.20) | 0.054 |
| The severity of COVID-19 | 0.65 (0.27-1.57) | 0.338 |
| Age | 1.53 (0.76-3.07) | 0.234 |
| Gender | 0.75 (0.36-1.54) | 0.427 |
| Donor-recipients gender matched | 0.87 (0.39-1.92) | 0.726 |
| Donor type | 1.11 (0.46-2.69) | 0.815 |
| Donor-recipient blood group matched | 0.56 (0.27-1.17) | 0.122 |
| HCT-CI before HSCT | 3.36 (1.46-7.75) | 0.004 |
| MNC counts in graft | 1.18 (0.59-2.34) | 0.640 |
| CD34^+^ cell counts in graft | 0.92 (0.46-1.81) | 0.798 |
| Lymphocytes at COVID-19 diagnosis | 0.83 (0.42-1.64) | 0.581 |
| Time from allo-HSCT to COVID-19 | 0.84 (0.52-2.01) | 0.656 |
| **Thrombocytopenia after COVID-19** |  |  |
| The duration of COVID-19 | 1.19(0.36-3.88) | 0.779 |
| The severity of COVID-19 | 2.69 (0.82-8.83) | 0.102 |
| Age | 2.65 (0.70-9.97) | 0.151 |
| Gender | 0.87 (0.25-2.97) | 0.823 |
| Donor-recipients gender matched | 2.30 (0.70-7.55) | 0.168 |
| Donor type | 1.05 (0.23-4.85) | 0.951 |
| Donor-recipient blood group matched | 0.77 (0.23-2.63) | 0.677 |
| HCT-CI before HSCT | 1.12 (0.14-8.75) | 0.914 |
| MNC counts in graft | 0.54 (0.16-1.86) | 0.332 |
| CD34^+^ cell counts in graft | 1.22 (0.37-4.00) | 0.742 |
| Lymphocytes at COVID-19 diagnosis | 1.78 (0.52-6.08) | 0.358 |
| Time from allo-HSCT to COVID-19 | 0.67 (0.43-2.63) | 0.647 |
| **CMV disease after COVID-19** |  |  |
| The duration of COVID-19 | 66.85 (0.15-29164.63) | 0.175 |
| The severity of COVID-19 | 20.15 (2.43-167.36) | 0.005 |
| Age | 5.98(0.72-49.69) | 0.098 |
| Gender | 2.08 (0.46-9.27) | 0.339 |
| Donor-recipients gender matched | 0.45 (0.05-3.74) | 0.460 |
| Donor type | 0.04 (0.00-164.88) | 0.442 |
| Donor-recipient blood group matched | 0.22 (0.03-1.86) | 0.166 |
| HCT-CI before HSCT | 1.79 (0.22-14.86) | 0.590 |
| MNC counts in graft | 0.39 (0.08-2.03) | 0.265 |
| CD34^+^ cell counts in graft | 6.02 (0.73-50.02) | 0.096 |
| Lymphocytes at COVID-19 diagnosis | 0.40 (0.08-2.04) | 0.268 |
| Time from allo-HSCT to COVID-19 | 0.55 (0.75-2.74) | 0.560 |
| **NRM after COVID-19** |  |  |
| The duration of COVID-19 | 9.87 (2.29-42.57) | 0.002 |
| The severity of COVID-19 | 22.77 (6.66-77.80) | < 0.0001 |
| Age | 1.87 (0.75-4.69) | 0.182 |
| Gender | 0.81 (0.32-2.03) | 0.649 |
| Donor-recipients gender matched | 0.69 (0.23-2.06) | 0.502 |
| Donor type | 0.53 (0.12-2.28) | 0.392 |
| Donor-recipient blood group matched | 0.44 (0.16-1.20) | 0.108 |
| HCT-CI before HSCT | 2.04 (0.60-6.94) | 0.257 |
| MNC counts in graft | 0.65 (0.27-1.59) | 0.343 |
| CD34^+^ cell counts in graft | 1.19 (0.49-2.86) | 0.706 |
| Lymphocytes at COVID-19 diagnosis | 0.42 (0.16-1.10) | 0.078 |
| Time from allo-HSCT to COVID-19 | 0.79 (0.33-2.16) | 0.523 |
| **OS after COVID-19** |  |  |
| The duration of COVID-19 | 3.87 (1.56-9.60) | 0.003 |
| The severity of COVID-19 | 11.99 (5.06-28.43) | < 0.0001 |
| Age | 1.07 (0.50-2.28) | 0.860 |
| Gender | 0.90 (0.41-1.96) | 0.784 |
| Donor-recipients gender matched | 0.62 (0.23-1.62) | 0.326 |
| Donor type | 0.37 (0.09-1.57) | 0.177 |
| Donor-recipient blood group matched | 0.76 (0.35-1.67) | 0.498 |
| HCT-CI before HSCT | 2.14 (0.74-6.19) | 0.161 |
| MNC counts in graft | 1.07 (0.50-2.28) | 0.859 |
| CD34^+^ cell counts in graft | 1.04 (0.49-2.21) | 0.924 |
| Lymphocytes at COVID-19 diagnosis | 0.40 (0.18-0.92) | 0.031 |
| Time from allo-HSCT to COVID-19 | 1.86 (0.58-4.09) | 0.382 |

**Abbreviations:** HCT-CI, Hematopoietic Cell Transplantation–Specific Comorbidity Index; MNC, mononuclear cell; PGF, poor graft function; CMV, cytomegalovirus; NRM, non-relapse mortality; OS, overall survival.

**Supplementary table 4. Multivariate analysis of risk factors for the 150-day clinical outcomes after COVID-19 infection**

| **Outcomes** | **HR (95% CI)** | ***P* value** |
| --- | --- | --- |
| **PGF after COVID-19** |  |  |
| Lymphocytes at COVID-19 diagnosis |  | 0.010 |
| ≤1.00 | 1 |  |
| >1.00 | 0.32 (0.13-0.76) |  |
| **Leukopenia after COVID-19** |  |  |
| HCT-CI before HSCT |  | 0.009 |
| <3 | 1 |  |
| ≥3 | 3.05 (1.32-7.08) |  |
| **NRM after COVID-19** |  | 0.032 |
| Lymphocytes at COVID-19 diagnosis |  |  |
| ≤1.00 | 1 |  |
| >1.00 | 0.35 (0.13-0.91) |  |
| **OS after COVID-19** |  |  |
| Lymphocytes at COVID-19 diagnosis |  | 0.004 |
| ≤1.00 | 1 |  |
| >1.00 | 0.30 (0.13-0.68) |  |

**Abbreviations:** HCT-CI, Hematopoietic Cell Transplantation–Specific Comorbidity Index; PGF, poor graft function; NRM, non-relapse mortality; OS, overall survival.

**Supplementary table 5.** **Characteristics between patients**

**with and without COVID-19**

| **Characteristics** | **COVID-19 (n = 179)** | **Without COVID-19 (n=179)** |
| --- | --- | --- |
| Gender, male/female, n (%) | 108 (60.3)/71 (39.7) | 99 (55.3)/80 (44.7) |
| Age(years), range | 40 (5-75) | 39 (10-63) |
| Underlying disease, n (%) |  |  |
| AL | 131 (73.2) | 139 (77.7) |
| MDS | 24 (13.4) | 20 (11.2) |
| AA | 5 (2.8) | 5 (2.8) |
| Lymphoma | 10 (5.6) | 13 (7.3) |
| Others | 9 (5.0) | 2 (1.1) |
| Donor match, n (%) |  |  |
| HLA-matched sibling donor | 31 (17.3) | 20 (11.2) |
| HLA-matched unrelated donor | 6 (3.4) | 4 (2.2) |
| Haploidentical related donor | 142 (79.3) | 155 (86.6) |
| Blood group matched, n (%) |  |  |
| Matched | 103 (57.5) | 100 (55.9) |
| Minor mismatched | 30 (16.8) | 35 (19.6) |
| Major mismatched | 46 (25.7) | 44 (24.6) |
| HCT-CI before HSCT, n (%) |  |  |
| 0 (low risk) | 123 (68.7) | 102 (57.0) |
| 1-2 (intermediate risk) | 41 (22.9) | 51 (28.5) |
| ≥3 (high risk) | 15 (8.4) | 26 (14.5) |
| Median counts of MNC in graft, range (× 10^8^/kg) | 9.51 (3.83-19.90) | 8.65 (5.60-14.90) |
| Median counts of CD34^+^ cell in graft, range (× 10^6^/kg) | 3.08 (0.48-17.20) | 2.45 (1.01-10.60) |
| Conditioning, n (%) |  |  |
| Chemotherapy-based | 168 (93.9) | 170 (95.0) |
| TBI-based | 11 (6.1) | 9 (5.0) |

**Abbreviations:** AL, acute leukemia; MDS, myelodysplastic syndromes; AA, aplastic anemia; HLA, human leukocyte antigen; HCT-CI, Hematopoietic Cell Transplantation–Specific Comorbidity Index; MNC, mononuclear cell; TBI, total body irradiation.

*P* < 0.05, *; *P* < 0.01, **.

**Supplementary table 6. Cause of death in the group without COVID-19**

| **Cause of death** | **n (%)** |
| --- | --- |
| Infection besides of COVID-19 | 4 (36.4) |
| CMV pneumonia | 2 (18.2) |
| Severe pneumonia of unknown etiology | 2 (18.2) |
| Relapse | 4 (36.4) |
| PGF | 1 (9.1) |
| aGVHD | 2 (18.2) |

**Abbreviations:** PGF, poor graft function; CMV, cytomegalovirus; aGVHD, acute graft versus disease.
